# Identifying the optimal dose of cannabidiol by intrabuccal administration in Kramnik (C3HeB/FeJ) mice

**DOI:** 10.1002/ame2.12569

**Published:** 2025-02-17

**Authors:** Oluwadara Pelumi Omotayo, Siyethemba Bhengu, Kobus Venter, Yolandy Lemmer, Shayne Mason

**Affiliations:** ^1^ Human Metabolomics, Faculty of Natural and Agricultural Sciences North‐West University Potchefstroom South Africa; ^2^ Preclinical Drug Development Platform, Faculty of Health Sciences North‐West University Potchefstroom South Africa; ^3^ Future Production and Chemicals Council for Scientific and Industrial Research (CSIR) Pretoria South Africa

**Keywords:** brain, C3HeB/FeJ mice, cannabidiol (CBD), dosage, intrabuccal administration, LC–MS/MS

## Abstract

**Background:**

Cannabidiol (CBD) has numerous therapeutic properties, and is used to treat neurological conditions, such as neuroinflammation. However, the optimal dose of CBD to penetrate the brain requires further investigation. The primary aim of this study was to use a mouse model and the intrabuccal route for CBD administration to determine the optimal dose at which CBD can penetrate the brain. The secondary aim was to determine whether sex is a confounding factor.

**Methods:**

Thirty adult Kramnik mice, divided equally into three groups, were administered CBD oil intrabuccally at three doses—10, 20, and 30 mg/kg, euthanized 6 h later, and whole brain, urine, and blood samples were collected. Liquid chromatography with tandem mass spectrometry was used to analyze the collected samples.

**Results:**

CBD and its three metabolites—7‐carboxy cannabidiol (7‐COOH‐CBD), 7‐hydroxy cannabidiol (7‐OH‐CBD) and 6‐hydroxy cannabidiol (6‐OH‐CBD), were identified and quantified in all samples. The 10 and 20 mg/kg doses of CBD produced similar results in the brain, but the group given the 10 mg/kg dose had the least variation. The 30 mg/kg dose yielded the highest abundance of CBD and its metabolites in all samples, but also the greatest variation. Sex only became a confounding factor at 30 mg/kg.

**Conclusions:**

This study shows that the intrabuccal route of CBD administration is reliable and the 10 mg/kg dose of CBD is recommended in mice because there were good CBD metabolite concentrations in all samples, with the least variation among the doses, and sex was not a confounder at 10 mg/kg.

## Graphical abstract

Cannabidiol (CBD) has numerous therapeutic properties, and is used to treat neurological conditions, such as neuroinflammation. However, the optimal dose of CBD to penetrate the brain requires further investigation. The primary aim of this study was to use a mouse model and the intrabuccal route for CBD administration to determine the optimal dose at which CBD can penetrate the brain. The secondary aim was to determine whether sex is a confounding factor. Thirty adult Kramnik mice, divided equally into three groups, were administered CBD oil intrabuccally at three doses—10, 20, and 30 mg/kg, euthanized 6 h later, and whole brain, urine, and blood samples were collected. Liquid chromatography with tandem mass spectrometry was used to analyze the collected samples. CBD and its three metabolites—7‐carboxy cannabidiol (7‐COOH‐CBD), 7‐hydroxy cannabidiol (7‐OH‐CBD) and 6‐hydroxy cannabidiol (6‐OH‐CBD), were identified and quantified in all samples. The 10 and 20 mg/kg doses of CBD produced similar results in the brain, but the group given the 10 mg/kg dose had the least variation. The 30 mg/kg dose yielded the highest abundance of CBD and its metabolites in all samples, but also the greatest variation. Sex only became a confounding factor at 30 mg/kg. This study shows that the intrabuccal route of CBD administration is reliable and the 10 mg/kg dose of CBD is recommended in mice because there were good CBD metabolite concentrations in all samples, with the least variation among the doses, and sex was not a confounder at 10 mg/kg.

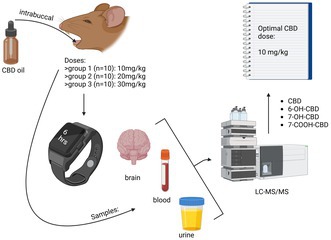

## 1 INTRODUCTION

Cannabidiol (CBD) is a naturally occurring phytocannabinoid derived from *Cannabis sativa* that is gaining popularity as a natural, plant‐based therapeutic drug.^1^ CBD acts as a secondary metabolite that can influence human biological functions.^2^, ^3^, ^4^ Numerous studies focus on CBD and its therapeutic potential in various neurological and neuropsychiatric disorders,^5^, ^6^, ^7^, ^8^ and its anti‐inflammatory properties have been reported in various studies.^9^, ^10^, ^11^ In 2018, Epidiolex® became the first commercial prescription CBD product approved by the Food and Drug Administration (FDA) for the management and treatment of seizures associated with Lennox–Gastaut syndrome and Dravet syndrome.^12^ The growing interest in the medicinal use of CBD in the field of neurology calls for more studies that examine the biochemistry behind CBD and its metabolites in different biological matrices. Moreover, CBD crosses the blood–brain barrier (BBB) through passive diffusion due to its lipophilicity and low molecular weight.^13^ Hence, for safe therapeutic use for neurological purposes, there is a need to identify the optimal dose at which CBD can penetrate the brain and be readily available, of which there is a paucity of information.

Studies have shown that the most common route of CBD administration is oral ingestion, which results in about 6%–10% bioavailability of CBD in the system. However, this route results in a much lower bioavailability of CBD specifically in the brain.^14^ This could be attributed to the extensive first‐pass metabolism that causes most of the active metabolites of CBD to be excreted. Hložek et al.^15^ compared oral and subcutaneous routes of CBD administration and observed that the oral route delivered a higher concentration of CBD to the brain than the subcutaneous route (>200 and <50 ng/g after oral and subcutaneous administration respectively). In the study by Deiana et al.^16^ in which the oral and intraperitoneal routes were investigated, it was observed that the oral route resulted in a higher concentration of CBD in the brain (8.6 μg/g). Hence, compared to the oral route, both the subcutaneous and intraperitoneal routes of CBD administration yield less bioavailability of CBD in the brain. However, the first‐pass effect associated with the oral route remains a major challenge. For example, Sallam et al.^17^ administered several cannabinoids to mice using oral gavage, but reported a delay in the time to the peak concentrations of these cannabinoids in plasma, brain, and adipose tissue.^17^

A less utilized route of CBD administration in research is the intrabuccal route—in the cheek or under the tongue (not swallowed) to allow diffusion through the oral mucosa; yet this route has been linked to better bioavailability of drugs (especially those with low oral bioavailability) in the system and quicker onset of action.^18^, ^19^ This is attributed to the fact that CBD administered via the intrabuccal route bypasses the digestive enzymes of the stomach and intestinal tract; hence, bypasses first‐pass metabolism.

To date, few studies have reported the bioavailability of CBD in biological matrices after administration using the intrabuccal route. This study is among the few that propose that CBD, when administered intrabuccally, can penetrate the brain faster (within 6 h of administration) than the oral route, and at acceptable quantifiable levels to show good bioavailability. Therefore, the primary aim of this study was to determine the optimal dose of CBD that can penetrate the brain after intrabuccal administration in Kramnik (C3HeB/FeJ) mice, with the secondary objective of determining if sex is a confounding factor. To our knowledge, this study is the first to demonstrate intrabuccal administration of CBD using the Kramnik mice model.

## 2 METHODS

### 2.1 Chemicals and reagents

The CBD oil (RELEAF 3000 mg—a replica of Epidiolex) used in this study was supplied by Afriplex Pharmaceutical Company in South Africa. Reference standards (purity >99%) for CBD and its three metabolites—7‐carboxy cannabidiol (7‐COOH‐CBD), 7‐hydroxy cannabidiol (7‐OH‐CBD) and 6‐hydroxy cannabidiol (6‐OH‐CBD), were supplied by the LGC Standards Group. A solution of 3‐phenylbutyric acid/nonadecanoic acid was used as an internal standard and purchased from Merck/Sigma‐Aldrich. Methanol, acetonitrile and formic acid (98%) were purchased from Merck/Sigma‐Aldrich (Germany) and were of LC–MS grade. Ultrapure water was obtained using a Millipore system, Milli Q (Merck). The mobile phase A consisted of water and 0.1% formic acid, while the mobile phase B consisted of acetonitrile and methanol (70:30) with 0.1% formic acid. All other solvents used for sample preparation were analytical grade. Methanol was used to prepare the stock solutions of the reference standards as well as their linear calibration dilution series.

### 2.2 Animals

Male and female Kramnik (C3HeB/FeJ) mice (JAX stock # 000658; The Jackson Laboratory) were bred and raised until they reached adulthood (8 weeks old), weighing between 17 and 26 g. The Kramnik mouse model was chosen for this CBD study because Kramnik mice are susceptible to infection by *Mycobacterium tuberculosis* (*Mtb*) and follow‐up research within our group will be testing the therapeutic effects of CBD in *Mtb*‐infected mice. Hence, this study is necessary to determine the optimal dose of CBD via the intrabuccal route of administration before proceeding to the next (infection) stage of the larger project. All experimental animals were reared and housed at the North‐West University (NWU) vivarium (SAVC reg. no. FR15/13458; SANAS GLP compliance no. G0019; AAALAC accreditation international file #1717) of the NWU Pre‐Clinical Drug Development Platform. The animals (total of 30) were housed in well‐ventilated cages under standard conditions. Five mice were housed per Tecniplast GM500 individually ventilated cage (391 mm width, 199 mm depth, and 160 mm height). The animal room received HEPA‐filtered air under positive pressure and at 23 air changes per hour. The room temperature was maintained at 22 ± 2°C, relative humidity was 55 ± 15%, and the light/dark cycle was set at 12 h each. A sterile corn cob was used for bedding that was supplemented with paper towels for nesting material and PVC pipes for environmental enrichment. Mice had ad libitum access to water and food and were weighed and evaluated prior to any intervention for baseline clinical signs.

The mice were allowed to acclimate for 5 days prior to the start of the experiment or the administration of the CBD oil (RELEAF 3000 mg). All procedures performed on the animals were in accordance with the ethics code for research, training, and testing in South Africa and complied with national legislation. The care of the mice was done according to the animal ethics regulations. The animals were monitored throughout the period of CBD administration and on an hourly basis after CBD administration prior to sample collection. The study protocol and experimental design received approval from the North‐West University Animal Care, Health, and Safety Research Ethics Committee (NWU‐AnimCareREC) with the approval number: NWU‐00785‐23‐A5.

The mice were assigned to three treatment groups using a block randomization technique, with each group consisting of 10 mice (five male and five female). They were fasted overnight before CBD administration, to increase CBD absorption, as was done by Sallam et al.^18^ In the morning, each mouse received an intrabuccal dose of CBD, as follows:

- Group 1: 10 mice (5 male and 5 female) received 10 mg/kg CBD.
- Group 2: 10 mice (5 male and 5 female) received 20 mg/kg CBD.
- Group 3: 10 mice (5 male and 5 female) received 30 mg/kg CBD.

The 10 and 20 mg/kg doses of CBD are supported by literature (0.9–13.9 mg/kg inhaled CBD in rats by Schwotzer et al.^20^ and 10 mg/kg vaporized CBD in rats by Hložek et al.^15^), and the next logical increment to 30 mg/kg is considered the high dose in this study. Each mouse was anesthetised with 5% isofluorane and 100% oxygen using a VetFlow™ anesthesia system before intrabuccal administration of CBD to limit stress and ensure an accurate dose. Before the experiment, this intrabuccal route of administration was first tested by pipetting 10 μL of 15% methylene blue into the cheeks of three control mice. During the procedure, each anesthetised mouse was held in dorsal recumbency, the required volume of methylene blue was aspirated, and the cheek was pulled from the bottom jaw with the pipette tip while simultaneously dispensing the dose into what is deemed the buccal mucosa. The blue color of the dye was observed to remain in the cheek; therefore, this route of administration was considered acceptable. After the intrabuccal administration of CBD, all experimental animals were placed in metabolic cages for the collection of urine samples. In rats, CBD has been reported to take up to 6 h (*T*_max_) to reach the maximum concentration (*C*_max_), especially when administered orally.^20^ Six hours after CBD administration, the animals were exsanguinated by transection of the carotid arteries and jugular veins, after which blood and whole brain samples were collected. The brain was rinsed in ice‐cold phosphate‐buffered saline (PBS) to remove blood from the brain sample. All samples were snap‐frozen using liquid nitrogen and stored at −80°C until analysis.

### 2.3 Preparation of analytical standard solutions

As was previously done by other groups, such as that of Schwotzer et al.,^20^ the stock solutions of the purchased analytical standards of CBD and its metabolites were diluted in methanol to obtain working solutions at concentrations of 1, 10 and 100 μg/mL for 6‐OH‐CBD, 2.5, 10, 25 and 50 μg/mL for 7‐OH‐CBD, 2.5, 5, 10 and 25 μg/mL for 7‐COOH‐CBD, and 2.5, 5 and 25 μg/mL for CBD. A concentration of 50 μg/mL of 3‐phenylbutyric acid/nonadecanoic acid in water was prepared for use as an internal standard solution^21^ (the reliability and stability of these compounds in biological matrices made them viable options for use as internal standards). The CBD oil product (RELEAF 3000 mg, supplied by Afriplex) had a CBD content per 30 mL of 3374.75 mg, or a specific gravity of 0.927 (0.927 g/mL). Hence, 1 mL contained 927 mg of CBD, or 0.927 mg/μL. For an average weight of ~20 g per mouse, a 10 mg/kg dose (or 0.2 mg/20 g) required 0.1854 μL of the CBD product; a 20 mg/kg dose (or 0.4 mg/20 g) required 0.3708 μL of the CBD product; and a 30 mg/kg dose (or 0.6 mg/20 g) required 0.5562 μL of the CBD product. These volumes are too low to accurately administer, so they were diluted 10 times with sunflower oil (vehicle) so that the dosage volumes required were 1.85 μL for the low dose (10 mg/kg), 3.71 μL for the medium dose (20 mg/kg), and 5.56 μL for the high dose (30 mg/kg).

### 2.4 Liquid chromatography–tandem mass spectrometry (LC–MS/MS)

Liquid chromatography analysis was performed on the Agilent 6470 LC system with electrospray ionization in positive ionization mode. The LC system consisted of a thermostated autosampler, a binary pump, an online degasser, and a column compartment. All samples were analyzed using the Waters Acquity UPLC BEH C18 column (1.7 μm particle size; 2.1 × 50 mm) (Fischer Scientific, Germany). Mobile phase A was composed of water and 0.1% formic acid, while acetonitrile, methanol (70:30), and 0.1% formic acid were mobile phase B. The column oven was set at 30°C, the injection volume was 1 μL, and the gradient elution was carried out at a flow rate of 0.2 mL/min. Initial gradient conditions were 99% A (0.1% formic acid in water [v/v]) and 1% B (0.1% formic acid in acetonitrile and methanol [v/v]). The initial composition of 1% B was maintained for 4 min; it increased from 1% to 90% and was maintained at 90% for 3 min before increasing to 100% and being maintained at 100% for 2 min before returning to the initial conditions for 1 min. The chromatographic conditions were optimized by analyzing the standard solutions. The detection and quantification of all analytes were performed using multiple reaction monitoring modes (MRM) due to the high sensitivity and selectivity (Table 1). For each of the analytes, one precursor ion and one or two MRM transitions were set up, with the less abundant product ion being monitored as qualifier ions, and the more abundant product ion as quantifier ions for confirmation. Table 2 shows the parameters for the quantification of CBD in the sample.

**TABLE 1:** LC–MS/MS settings.

	Brain	Urine	Blood
Type	Multiple reaction monitoring (MRM) at two transitions	Multiple reaction monitoring (MRM) at two transitions	Multiple reaction monitoring (MRM) at two transitions
Primary transition	Quantitation	Quantitation	Quantitation
Secondary transition	Qualitative confirmation	Qualitative confirmation	Qualitative confirmation
Range of quantitation	0.1–980 ng/mL	1–6050 ng/mL	0.1–11 864 ng/mL
Ionization	Electrospray ionization	Electrospray ionization	Electrospray ionization

**TABLE 2:** LC–MS/MS parameters for the identification and quantification of CBD and its metabolites.

Analyte	Retention time (min)	Dwell time (ms)	Precursor ion (m/z)	Product ion (m/z)	Fragmentor	Collision energy (V)	Ion polarity
CBD	6.91	50	315.2	259.1	90	20	+
7‐OH‐CBD	6.40	50	331.2	313.2	95	12	+
6‐OH‐CBD	6.06	50	137.0	93.1	76	16	+
7‐COOH‐CBD	6.37	50	345.2	327.2	100	16	+

Abbreviations: 6‐OH‐CBD, 6‐hydroxy cannabidiol; 7‐COOH‐CBD, 7‐carboxy cannabidiol; 7‐OH‐CBD, 7‐hydroxy cannabidiol; CBD, cannabidiol.

### 2.5 Calibration model

Calibration curves were obtained from the peak‐area ratio of each analyte. The ratio (*y*‐axis) was plotted against the analyte concentration (*x*‐axis) to generate the standard curves by least squares using a weighed linear regression model. Curves with five concentrations were best fitted by linear least‐squares regression across the linear dynamic range, with a coefficient of determination (*R* ^2^) of 0.99. The optimized method was found to be selective and specific, and no interference peaks were observed in the blank samples at the retention times of the target compounds.

### 2.6 Sample preparation

#### 2.6.1 Whole brain samples

**FIGURE 1:**
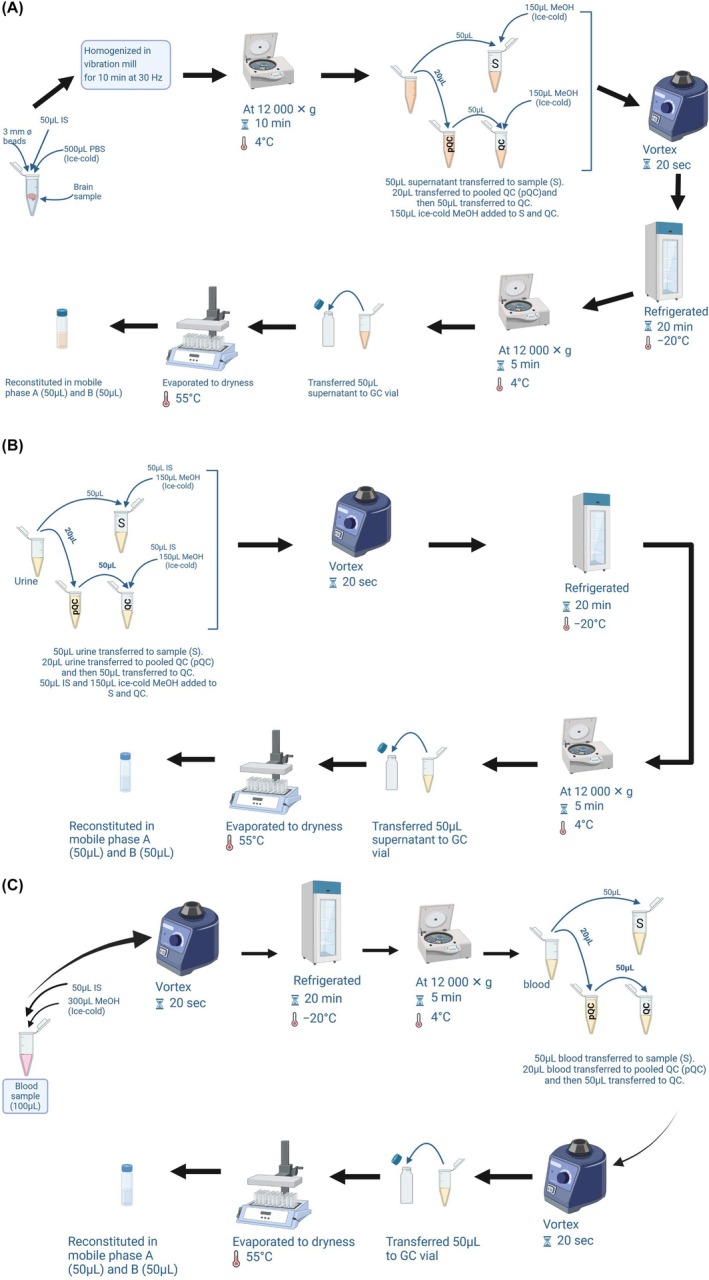
Procedure for sample preparation: (A) brain, (B) urine, (C) blood (Figure created with Biorender.com).

Following the protocol of Schwotzer et al.,^20^ 3 mm tungsten beads were added to 100 mg of whole brain samples, along with 500 μL of ice‐cold PBS (to submerge the sample) and 50 μL of IS solution. The samples were homogenized for 10 min at 30 Hz in a vibration mill (Figure 1A). The sample was centrifuged at 12 000 *g* for 10 min, followed by the separation of the homogenates from the beads and debris. Following centrifugation, 20 μL supernatant was collected from each sample to make a pooled quality control sample (pQC), from which 10 aliquots of pQCs (50 μL each) were obtained and prepared as per other samples. To remove proteins, a 50 μL volume of homogenate was collected and 150 μL of ice‐cold methanol was added, the mixture was vortexed for 20 s and refrigerated for 20 min at −20°C, followed by centrifugation at 12 000 *g* for 5 min at 4°C. The sample supernatant was transferred to a 2 mL glass GC tube, evaporated to dryness under a stream of nitrogen gas in a heating block set to 55°C, and then reconstituted in mobile phases A and B (50 μL each) before LC–MS/MS analysis.

#### 2.6.2 Urine samples

As illustrated in Figure 1B, 20 μL was taken from each urine sample to make a pQC sample, from which 10 aliquots of pQCs (50 μL each) were obtained and prepared as per other samples. A volume of 50 μL of urine samples and pQCs was transferred to a 2 mL microcentrifuge tube with 50 μL IS solution, followed by the addition of 150 μL of ice‐cold methanol. The mixture was vortexed for 20 s and stored at −20°C for 20 min. The samples were centrifuged at 12 000 *g* for 5 min at 4°C to obtain the supernatant which was transferred to a 2 mL glass GC vial and evaporated to dryness under a stream of nitrogen gas in a heating block set to 55°C. The residue was later reconstituted in mobile phases A and B (50:50 v/v) before LC–MS/MS analysis.

#### 2.6.3 Blood samples

As illustrated in Figure 1C, 300 μL of methanol was added to 100 μL of blood samples followed by the addition of 50 μL of IS solution to the mixture, which was vortexed for 20 s and stored at −20°C for 20 min. The solution was centrifuged at 12 000 *g* for 10 min at 4°C and the supernatants were obtained. Twenty micolitres was obtained from each sample to make a pQC sample, from which 10 aliquots of pQCs (50 μL each) were obtained, and these were vortexed again for 20 s. A volume of 50 μL of the samples and pQCs was transferred to a 2 mL glass GC vial, evaporated to dryness under a stream of nitrogen gas in a heating block set to 55°C, reconstituted in mobile phases A and B (50:50 v/v), and analyzed in the LC–MS/MS system.

### 2.7 Statistical analysis

The concentrations of CBD and its metabolites in the samples were compared between doses (10, 20, and 30 mg/kg) using Microsoft Excel 2021, and illustrated using MetaboAnalyst (6.0). Student's *t* test was done to determine statistical significance (*p* < 0.05).

## 3 RESULTS

### 3.1 Purity of CBD product

Before the analysis of samples, an evaluation was done on the purity of the CBD product (RELEAF 3000 mg) used in this study. No traces of the delta‐8 and delta‐9 forms of THC were detectable in the CBD product. In terms of quantitative purity, the CBD product was found to have a >96% purity for CBD. The ~4% ‘other’ constituents are probably due to the strawberry flavoring chemicals advertised for this CBD product.

### 3.2 Distribution of CBD and its metabolites in the brain

**FIGURE 2:**
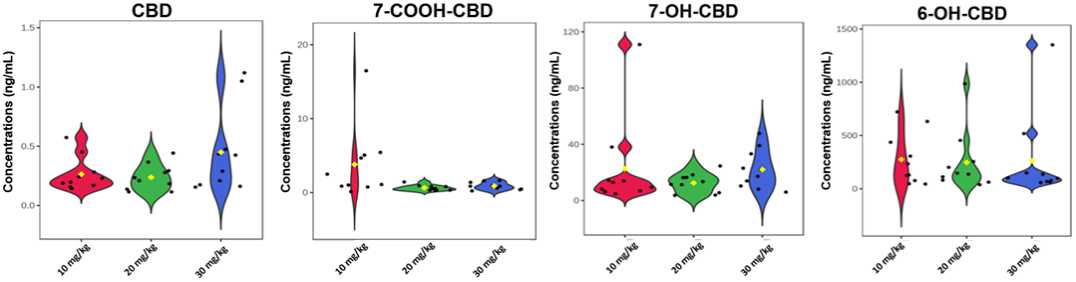
Violin plots of CBD and its three metabolites (7‐COOH‐CBD, 7‐OH‐CBD and 6‐OH‐CBD) in the brain 6 h after intrabuccal administration of a low (10 mg/kg), medium (20 mg/kg) and high (30 mg/kg) dose of CBD. 6‐OH‐CBD, 6‐hydroxy cannabidiol; 7‐COOH‐CBD, 7‐carboxy cannabidiol; 7‐OH‐CBD, 7‐hydroxy cannabidiol; CBD, cannabidiol.

Following CBD administration, no signs of distress were observed in the animals and no mortality was recorded; therefore, the CBD doses were well tolerated by the mice. At 6 h, the median concentrations of 0.21 ± 0.14, 0.22 ± 0.11, and 0.36 ± 0.36 ng/mL were recorded for CBD in the brains of the mice given 10, 20, and 30 mg/kg CBD, respectively (Figure 2). Hence, the 10 and 20 mg/kg doses of CBD yielded approximately the same amount of CBD, and the same variation, in the brain, whereas the 30 mg/kg dose yielded a 1.6‐fold increase in median CBD concentration, but with a 3‐fold increase in variation, in the brain. The median concentrations of 7‐OH‐CBD recorded in the brain of mice administered CBD intrabuccally were 11.05 ± 32.5, 12.46 ± 6.79, and 19.64 ± 13.97 ng/mL for the 10, 20, and 30 mg/kg doses of CBD, respectively (Figure 2). Hence, the CBD metabolite 7‐OH‐CBD followed a similar trend in median concentration as CBD—similar amounts at the low and medium dose, and a 1.6‐fold increase in concentration at the 30 mg/kg dose, but the variation of 7‐OH‐CBD was lowest at 20 mg/kg and highest at 10 mg/kg. At 6 h, the median concentrations of 7‐COOH‐CBD recorded in the brains of mice administered CBD intrabuccally were 1.79 ± 4.88, 0.55 ± 0.36, and 0.86 ± 0.5 ng/mL for the 10, 20, and 30 mg/kg CBD doses, respectively (Figure 2). Hence, the 10 mg/kg dose of CBD yielded the highest concentrations of 7‐COOH‐CBD in the brain, whereas the 20 and 30 mg/kg doses of CBD yielded up to 3‐fold less 7‐COOH‐CBD in the brain, but the variation was greatest at 10 mg/kg. Lastly, for 6‐OH‐CBD in the brain of the mice, there was 181.65 ± 245.9, 141.54 ± 286.01, and 100 ± 406.3 ng/mL recorded for the 10, 20, and 30 mg/kg doses of CBD, respectively (Figure 2).

**FIGURE 3:**
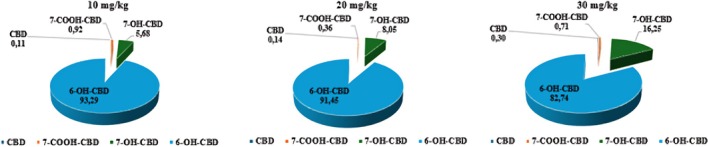
Distribution of the concentrations of CBD and its three metabolites (7‐COOH‐CBD, 7‐OH‐CBD and 6‐OH‐CBD) in the brain. 6‐OH‐CBD, 6‐hydroxy cannabidiol; 7‐COOH‐CBD, 7‐carboxy cannabidiol; 7‐OH‐CBD, 7‐hydroxy cannabidiol; CBD, cannabidiol.

In terms of relative amounts, 6‐OH‐CBD was the most dominant CBD metabolite in the brain with 93.3%, 91.5% and 82.7% for the 10, 20, and 30 mg/kg CBD doses, respectively (Figure 3). 7‐OH‐CBD was the next most abundant CBD metabolite in the brain with 5.7%, 8.1% and 16.3% for the 10, 20, and 30 mg/kg doses of CBD, respectively (Figure 3). Both CBD and its metabolite 7‐COOH‐CBD constituted less than 1% of the total distribution in the brain. From the independent *t*‐tests performed, no statistically significant differences (*p* > 0.05) were observed between the doses for CBD, 6‐OH‐CBD, 7‐OH‐CBD and 7‐COOH‐CBD in the brain (Table 3).

**TABLE 3:** The statistically significant differences between sex and doses in the brain, urine, and blood of mice recorded 6 h after CBD dosage.

Groups compared	*p*‐Values (brain)				*p*‐Values (urine)				*p*‐Values (blood)			
	CBD	7‐OH‐CBD	7‐COOH‐CBD	6‐OH‐CBD	CBD	7‐OH‐CBD	7‐COOH‐CBD	6‐OH‐CBD	CBD	7‐OH‐CBD	7‐COOH‐CBD	6‐OH‐CBD
10 mg/kg Male vs. female	0.87	0.23	0.43	0.26	0.60	0.23	0.41	0.83	0.47	0.86	0.73	0.16
20 mg/kg Male vs. female	0.83	0.56	0.99	0.53	0.46	0.90	0.73	0.16	0.16	0.37	0.34	0.12
30 mg/kg Male vs. female	0.22	0.003*	0.13	0.19	0.34	0.20	0.67	0.80	0.42	0.14	0.89	0.038*
10 vs. 20 mg/kg	0.65	0.35	0.06	0.82	0.53	0.27	0.91	0.45	0.19	0.13	0.40	0.24
10 vs. 30 mg/kg	0.14	0.97	0.08	0.93	0.92	0.22	0.62	0.95	<0.001*	<0.001*	0.27	<0.001*
20 vs. 30 mg/kg	0.09	0.07	0.21	0.92	0.60	0.12	0.58	0.56	0.001*	<0.001*	0.44	0.07

Abbreviations: 6‐OH‐CBD, 6‐hydroxy cannabidiol; 7‐COOH‐CBD, 7‐carboxy cannabidiol; 7‐OH‐CBD, 7‐hydroxy cannabidiol; CBD, cannabidiol.

* Statistically significant (*p* < 0.05) result.

### 3.3 Distribution of CBD and its metabolites in the urine

**FIGURE 4:**
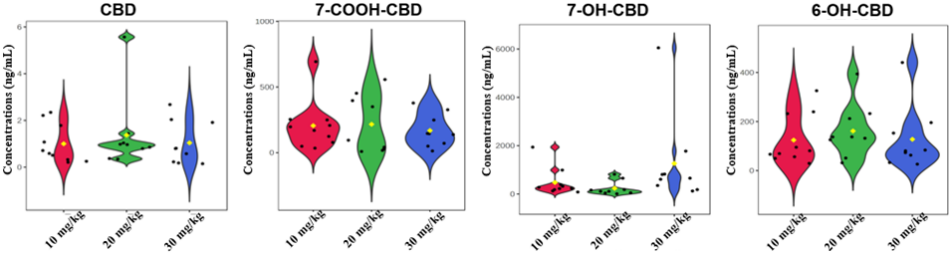
Violin plots of CBD and its three metabolites (7‐COOH‐CBD, 7‐OH‐CBD and 6‐OH‐CBD) in the urine 6 h after intrabuccal administration of a low (10 mg/kg), medium (20 mg/kg) and high (30 mg/kg) dose of CBD. 6‐OH‐CBD, 6‐hydroxy cannabidiol; 7‐COOH‐CBD, 7‐carboxy cannabidiol; 7‐OH‐CBD, 7‐hydroxy cannabidiol; CBD, cannabidiol.

The median concentrations of CBD recorded in urine samples ranged from 0.65 (10 mg/kg) to 0.97 ng/mL (3 mg/kg). Similar to the brain, the percentage concentration of 7‐OH‐CBD in the urine was higher than that of 7‐COOH‐CBD and CBD, with 7‐OH‐CBD median concentrations ranging from 109.39 to 654.14 ng/mL. The median concentration of 6‐OH‐CBD ranged between 75.25 and 137.29 ng/mL, while the concentration of 7‐COOH‐CBD observed in mice was not as high as that of 7‐OH‐CBD and ranged from 95.26 to 183.13 ng/mL. 7‐COOH‐CBD has been reported to be excreted primarily in feces and less in urine, which explains why its concentration was not as high as that of 7‐OH‐CBD.^20^ At 10 mg/kg, CBD and its metabolites were observed to be in sufficient abundance—equivalent to the 20 and 30 mg/kg CBD doses (Figure 4).

### 3.4 Distribution of CBD and its metabolites in blood samples

**FIGURE 5:**
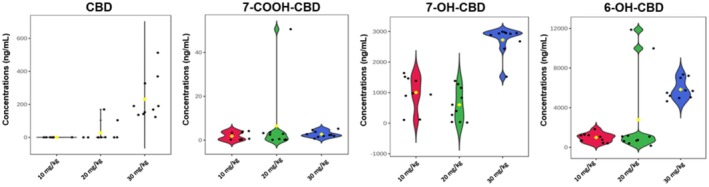
Violin plots of CBD and its three metabolites (7‐COOH‐CBD, 7‐OH‐CBD and 6‐OH‐CBD) in the blood 6 h after intrabuccal administration of a low (10 mg/kg), medium (20 mg/kg) and high (30 mg/kg) dose of CBD. 6‐OH‐CBD, 6‐hydroxy cannabidiol; 7‐COOH‐CBD, 7‐carboxy cannabidiol; 7‐OH‐CBD, 7‐hydroxy cannabidiol; CBD, cannabidiol.

The concentrations of CBD and its metabolites observed in the blood samples were in the order 6‐OH‐CBD > 7‐OH‐CBD > 7‐COOH‐CBD > CBD. 6‐OH‐CBD was the most abundant metabolite in the blood with median concentrations ranging from 909.87 to 5595.42 ng/mL, 7‐OH‐CBD ranged between 500.65 and 2930.82 ng/mL and 7‐COOH‐CBD from 0.63 to 2.36 ng/mL. At 6 h, median concentrations of 0.58 ± 0.23, 0.43 ± 59.48, and 179.21 ± 129.03 ng/mL of CBD were recorded in the blood samples of mice administered 10, 20, and 30 mg/kg CBD, respectively (Figure 5). Thus, the 30 mg/kg dose of CBD produced the highest concentration of CBD in the blood, as expected; however, this high dose also yielded the greatest variation. Little or no differences were observed in the median CBD concentrations in the blood of the animals administered 10 and 20 mg/kg CBD, but the medium dose yielded some variation (half that of the high dose), while the low dose had negligible variation. It is noteworthy that low and medium doses of CBD were almost completely converted to its metabolites; however, at 30 mg/kg there were appreciable amounts of CBD circulating in the blood that were not metabolized.

Like the brain and urine samples, the concentrations of 7‐COOH‐CBD in the blood (0.63 ± 1.82, 1.51 ± 15.59 and 2.36 ± 1.43 ng/mL for the 10, 20, and 30 mg/kg dose of CBD, respectively) were low, compared to 6‐OH‐CBD and 7‐OH‐CBD. At the 10 mg/kg CBD dose, 6‐OH‐CBD (1057.57 ± 458.56 ng/mL) and 7‐OH‐CBD (976.67 ± 573.74 ng/mL) had similar median concentrations and variance. However, at the 20 and 30 mg/kg doses of CBD, 6‐OH‐CBD (909.87 ± 4326.99 ng/mL for the 20 mg/kg dose and 5595.42 ± 993.78 ng/mL for the 30 mg/kg dose) was approximately double the concentration of 7‐OH‐CBD (500.65 ± 532.38 ng/mL for the 20 mg/kg dose and 2930.82 ± 457.03 ng/mL for the 30 mg/kg dose), but with greater variation (Figure 5).

### 3.5 Sex differences

An independent *t*‐test was performed to compare the concentrations of CBD and its three metabolites between males and females for all treatment groups (Table 3). No statistically significant differences (*p* > 0.05) were found between males and females for CBD and its three metabolites in the blood, brains and urine of the mice administered with the 10 and 20 mg/kg doses of CBD. These findings are similar to those of Schwotzer et al.,^20^ where they reported no significant differences in the *C*_max_ of CBD in male and female Sprague–Dawley rats. However, at 30 mg/kg, a significant difference (*p* < 0.05) between males and females was observed in the concentrations of 7‐OH‐CBD recorded in the brain, as well as in the concentration of 6‐OH‐CBD in the blood, of the mice.

### 3.6 Quality of data

It should be noted that the analytical method used in this study is based on the method published by Schwotzer et al.^20^ Briefly, this method involves a protein crash using ice‐cold methanol, followed by LC–MS/MS analysis. We evaluated the repeatability of the experimental data by calculating the coefficient of variation (CV) values of the pQCs for the various matrices. The pQCs for the brain samples produced CV values of 79%, 92%, 82% and 106% for CBD, 6‐OH‐CBD, 7‐OH‐CBD, and 7‐COOH‐CBD, respectively. For the urine samples, the pQCs yielded CV values of 85%, 110%, 69%, and 64% for CBD, 6‐OH‐CBD, 7‐OH‐CBD and 7‐COOH‐CBD, respectively. For the blood samples, the pQCs yielded CV values of 73%, 73%, 59%, and 70% for CBD, 6‐OH‐CBD, 7‐OH‐CBD and 7‐COOH‐CBD, respectively. Hence, there is room for improvement in the quality of the data, which probably could be obtained by including a metabolite extraction method during sample preparation, rather than just a protein crash (ice‐cold methanol) step.

## 4 DISCUSSION

Despite the use of several delivery systems in the past for CBD administration, the issue of first‐pass metabolism, which is commonly faced, particularly with orally taken medications, persists. Hence, in this study, the intrabuccal route, which has been identified as one in which the drug administered bypasses first‐pass metabolism, was used.^19^ According to Nair et al.,^19^ the intrabuccal route of administration is a desirable choice for drug delivery into the systemic circulation as it bypasses first‐pass effects. The study by Koyi and Khan,^18^ who evaluated the intrabuccal route as one that offers controlled drug delivery over a long period in addition to avoiding first‐pass metabolism, also justifies this route.

In this study, a single‐dose administration of CBD at 10, 20, and 30 mg/kg, through the intrabuccal route, resulted in quantifiable levels of CBD in the brain after 6 h of administration. The detection of CBD at quantifiable levels even at a lower dose of 10 mg/kg implies that CBD was well absorbed without being broken down by the digestive enzymes. This corroborates the study of Patel et al.,^22^ which appraised the intrabuccal route and indicated that it reduces gastric degradation and provides a faster onset of action.

To the best of our knowledge, this is the first study to use the Kramnik mouse model to demonstrate intrabuccal delivery of CBD. The detection of CBD and its metabolites in the brain at quantifiable levels indicates that this route of administration is suitable. In contrast to 10 mg/kg, administration of 20 and 30 mg/kg CBD resulted in higher, but not statistically different concentrations of CBD and its metabolites in the brain, albeit with greater variations. This suggests that the lowest 10 mg/kg dose of CBD is sufficiently detectable in the brain, supporting the findings of Hložek et al.,^15^ which identified 10 mg/kg of CBD as a dosage detectable in the brain. The results from this study are also similar to the findings of Taylor et al.,^23^ which showed that an increase in CBD dose led to a rise in the concentration of this compound in the plasma.

Moreover, the quantifiable levels of CBD and its metabolites in the samples after 6 h in this study corroborate findings by Deiana et al.,^16^ in a study of the pharmacokinetic profile of CBD in rats after an acute single dose of CBD, of quantifiable levels of CBD in the brain 6 h after oral administration.

Furthermore, results of this study indicate that sex has no significant influence on the concentration and bioavailability of CBD in the brain of Kramnik mice at 10–20 mg/kg dosages. Few studies exist which examine the effect of sex on the concentration of CBD and its bioavailability in the brain, especially in mice. A study conducted by Briânis et al.^24^ who examined the effect of sex on CBD concentration in C57BL6/J mice confirmed that administration of CBD to both male and female C57BL6/J mice had similar effects (no statistically significant difference). Similarly, in rats, Rock et al.^25^ demonstrated that sex does not significantly influence the effects of CBD, indicating that CBD may be equally effective in males and females. Furthermore, Craft et al.^26^ observed that rats exposed acutely to vaporized CBD‐dominant cannabis extract did not exhibit any significant effects in either sex, meaning there were no differences in the response of each sex to the treatment.

The concentrations of 7‐OH‐CBD and CBD observed in the brain were similar, both rising with increasing doses of administered CBD. However, the opposite was observed for the concentration of 6‐OH‐CBD in the brain, as the lowest dose of CBD (10 mg/kg) resulted in a higher concentration of this metabolite in the brain. A higher concentration of 7‐COOH‐CBD was observed in mice administered a low dose of CBD (10 mg/kg) than the higher doses of 20 and 30 mg/kg (Figure 2). CBD is mainly metabolized to 7‐OH‐CBD, which is further converted to 7‐COOH‐CBD in the presence of the CYP2C19 enzyme.^27^ The high concentration of 7‐COOH‐CBD recorded in samples from mice administered 10 mg/kg CBD indicates that there was a rapid metabolism of CBD to 7‐OH‐CBD and further to 7‐COOH‐CBD at 6 h in these mice. This observation is similar to the study of Pigliasco et al.^27^ in which it was observed that 7‐COOH‐CBD was the metabolite with the highest concentration after the administration of various CBD‐containing formulations to epilepsy patients.^27^

For mice administered 20 and 30 mg/kg of CBD, high variation was seen in the concentrations of 7‐COOH‐CBD; this could have resulted from the greater variability of CBD concentrations observed in the brain and urine samples of mice administered with these doses. The abundance of 7‐OH‐CBD over CBD and 7‐COOH‐CBD observed in Figure 4 can be linked to the metabolism process; that is, CBD is first metabolized to 7‐OH‐CBD. Hence, the reduction in the concentration of CBD and increase in that of 7‐OH‐CBD. The low concentration of 7‐COOH‐CBD can also be linked to the experimental endpoint, as 6 h was probably too soon for the 7‐OH‐CBD to breakdown completely to 7‐COOH‐CBD. This is confirmed by the study of Turner et al.^28^ who reported a peak in the concentration of 7‐COOH‐CBD after 8 h of CBD administration; however, the study by Turner et al.^28^ was done in horses using oral and intravenous administration of CBD. Hence, there is a need for additional experimental studies to find the *T*_max_ of CBD and its metabolites in the brain of mice. 6‐OH‐CBD was also observed in higher concentrations than CBD in the brain, and this can also be associated with the breakdown and metabolism of CBD into its metabolites. While there are limited data on the prevalence of 6‐OH‐CBD in mice, this metabolite was indicated in the study of Chicoine et al.^29^ as a major primary metabolite in dogs administered with CBD‐enriched cannabis‐derived herbal extract.^29^

In the urine samples, except for 6‐OH‐CBD, the concentrations of other metabolites resulting from a 20 mg/kg dose were lower than those of 10 and 30 mg/kg doses, except for CBD, which had a higher concentration. As in the brain, 7‐OH‐CBD was the most abundant compared to the other three metabolites, with concentrations ranging from 109.388 ng/mL to as high as 654.139 ng/mL The high concentration of 7‐OH‐CBD seen in both urine and brain samples compared to other metabolites can be attributed to the experimental endpoint (6 h) and the rate of metabolism of CBD into 7‐OH‐CBD. It can be inferred that 6 h was probably insufficient for the metabolism of 7‐OH‐CBD to 7‐COOH‐CBD.

It has been reported that feces are the major route through which 7‐COOH‐CBD is excreted.^28^ This could also explain the reason why lower concentrations of 7‐COOH‐CBD were observed in the urine samples. When comparing males and females, for all treatment groups (Table 3), no significant differences were seen in the concentrations of the metabolites in the brain for mice administered the 20 and 30 mg/kg doses. This is similar to the findings of Schwotzer et al.,^20^ who also reported no significant difference in the metabolite concentration of Sprague–Dawley rats administered 10 mg/kg CBD. However, statistically significant differences (*p* < 0.05) in the concentrations of 7‐OH‐CBD and 7‐COOH‐CBD between the male and female mice were observed (*p* < 0.05) after administration of 30 mg/kg of CBD.

After 6 h, at a 30 mg/kg dose, the high concentration of CBD in the blood samples shows that CBD was still circulating in its original form and was not fully metabolized, thus indicating a saturation of the liver enzymes. We postulate that the liver is under stress at 30 mg/kg dose of CBD. Examining the liver over a period of chronic 30 mg/kg of CBD dosage will help to test this hypothesis. Similar observations were also recorded in the blood samples of mice administered 20 mg/kg of CBD, but to a lesser degree. Furthermore, significantly greater amounts of 7‐OH‐CBD and 6‐OH‐CBD were observed to be circulating in the blood samples of the mice; however, these metabolites were not as elevated in the brain as in the blood. This could be attributed to the limited transport across the BBB, as confirmed by the study of Wu et al.^30^ CBD metabolites increased as the dose increased, and so did the variation in their concentrations, and at 30 mg/kg sex starts becoming a confounder. Hence, from the above observations, 30 mg/kg dose does not produce repeatable/reliable results.

6‐OH‐CBD is the most abundant CBD metabolite in the brain and blood; however, 7‐OH‐CBD is more abundant in the urine. This is similar to the study of Pérez‐Acevedo et al.^31^ in which 7‐OH‐CBD was found to be the most abundant CBD metabolite in the urine of healthy human volunteers after the administration of medical cannabis, while the concentration of 6‐OH‐CBD was significantly lower in the urine. This can be attributed to a delay in the metabolism of 6‐OH‐CBD.

- Although the sample size of 30 mice was deemed adequate for this study (a power analysis was done for the experimental design—a total of seven cases per group would give us a probability of 97.1% that the study will detect a treatment difference at a two‐sided 0.05 significance level with an effect size of 0.87). However, a higher sample size would have yielded increased statistical power in the findings. This would have also addressed the unusual circumstances where biological samples could not be obtained from experimental animals, as was the case in this investigation where one mouse did not produce urine. In future research, augmenting the sample size would be advantageous to substantiate and extrapolate findings, as well as unveil more subtle differences or effects among the groups.
- This study found that mice receiving the highest dose of CBD drug (30 mg/kg) had a significant amount of CBD in their blood samples. This suggests that, at this concentration, CBD was not completely metabolized and was still circulating in the body, suggesting that the liver enzymes were saturated. Therefore, examining the liver would have yielded additional information regarding the concentration of CBD in this organ, thereby elucidating the effect of this dosage. Hence, in future studies, it is imperative to conduct an analysis of the liver to yield information into the impact of CBD at different concentrations on this organ. Moreover, in this study, sex became a confounding factor at the CBD dose of 30 mg/kg—our hypothesis is that the capacity of certain hepatic enzymes (e.g. cytochrome P450) differs between male and female mice. Hence, additional enzymatic studies within the liver should evaluate why higher doses of CBD cause differences between sexes in mice.
- The evaluation of the data quality revealed a relatively high CV ranging from 79% to 106% for brain samples, 64% to 110% for urine samples, and 59% to 73% for blood samples. This suggests a moderate to high level of variability in the measurements. This may be due to employing only the protein crash approach in the preparation of samples. However, it is worth noting that this study utilized a published and existing protocol.^20^ It is our recommendation that a metabolite extraction technique be incorporated into the sample preparation process in future studies, particularly for tissue samples such as whole brain samples.
- This study relied on a single timepoint measurement (*T*_max_—6 h), after which the experiment was terminated. Moreover, the *T*_max_ that was used in this study was based upon an experimentally derived point from a CBD brain study that utilized rats,^20^ not mice. Hence, the *T*_max_ used here may not have adequately recorded the complete dynamics of the bioavailability and pharmacokinetics and CBD and its metabolites in this mouse model. Also, utilizing only one time point restricts the understanding of the absorption, distribution, and elimination of CBD, specifically omitting significant peaks or changes in concentration (such as *C*_max_). Future research should incorporate numerous time periods into a longitudinal study to experimentally determine the *T*_max_ and *C*_max_ (i.e. optimal pharmacokinetic parameters) of CBD and its bioavailability in the brain of a mouse model.
- To the best of our knowledge, this study is the first investigation that has used the intrabuccal administration of CBD, and examined the brain, in a mouse model. Hence, there is no existing literature to serve as a basis for comparing the results of this study. The findings of this study may be influenced by the constraints inherent in a first‐generation study. Subsequent investigations can utilize this study as a foundation to authenticate and broaden the understanding of CBD's ability to penetrate the brain, and the intrabuccal route of delivery. Furthermore, conducting a similar study comparing the intrabuccal route of CBD administration to other routes of CBD administration (e.g., oral, subcutaneous, intravenous) would test the value of these results.

## 5 CONCLUSION

To determine the optimal dose of CBD, three doses of CBD (10, 20, and 30 mg/kg) were administered to male and female Kramnik mice. All administered doses were tolerable (with no adverse effects observed in the mice) and produced quantifiable amounts of CBD in the brain. The detection of CBD in the brains of mice in all the experimental groups shows that the drug penetrated the brain at all doses, and the quantifiable levels of CBD and metabolites at 10 mg/kg (the lowest dose) also indicate that the intrabuccal route is efficient for the delivery of CBD into the brain. This could be attributed to the fact that the administration of CBD intrabuccally allowed the drug to escape degradation by gastric enzymes in the stomach. As observed, CBD was metabolized into 6‐OH‐CBD, 7‐OH‐CBD, and 7‐COOH‐CBD, as these metabolites were detected and quantified in the brain, urine, and blood samples of the mice. In the brain and blood samples, 6‐OH‐CBD is the most abundant metabolized form of CBD, while 7‐OH‐CBD is more abundant in the urine. This can be attributed to the metabolism process and experimental endpoint (i.e., the metabolism of 6‐OH‐CBD was delayed in the brain and blood while that of 7‐OH‐CBD was delayed in the blood samples). The lower concentration of 7‐COOH‐CBD in the samples compared to other metabolites indicates that urine is not the major route through which the metabolite is excreted, and this is in line with other studies, as discussed. Except at the 30 mg/kg dose, no significant difference was observed in the concentrations of CBD when comparing male and female Kramnik mice. The detection of elevated CBD levels in the blood of mice given a dosage of 30 mg/kg CBD suggests that CBD was not completely metabolized within 6 h. This indicates that the liver enzymes may have reached saturation, implying that long‐term use of CBD at this dosage could potentially strain the liver. From this study, 10 mg/kg is recommended as it presents a more consistent concentration of CBD in the brain and other samples with less variation; conversely, the results produced at 30 mg/kg are not repeatable.

## AUTHOR CONTRIBUTIONS

**Oluwadara Pelumi Omotayo:** Data curation; formal analysis; investigation; methodology; writing – original draft. **Siyethemba Bhengu:** Data curation; investigation; methodology; writing – review and editing. **Kobus Venter:** Methodology; writing – review and editing. **Yolandy Lemmer:** Investigation; methodology; writing – review and editing. **Shayne Mason:** Conceptualization; formal analysis; funding acquisition; project administration; supervision; writing – review and editing.

## ETHICS STATEMENT

All procedures performed on the animals were in accordance with the ethics code for research, training, and testing in South Africa and complied with national legislation. The care of the mice was done according to the animal ethics regulations. The animals were monitored throughout the period of CBD administration and on an hourly basis after CBD administration prior to sample collection. The study protocol and experimental design received approval from the North‐West University Animal Care, Health, and Safety Research Ethics Committee (NWU‐AnimCareREC) with the approval number: NWU‐00785‐23‐A5.

## FUNDING INFORMATION

National Research Fund of South Africa (grant number: 137792).

## CONFLICT OF INTEREST STATEMENT

The authors declare no conflict of interest.
